# Video observations of treatment administration to children on antiretroviral therapy in rural KwaZulu-Natal

**DOI:** 10.1080/09540121.2016.1176674

**Published:** 2016-07-08

**Authors:** Bronwyne Coetzee, Ashraf Kagee, Ruth Bland

**Affiliations:** ^a^ Department of Psychology, Stellenbosch University, Stellenbosch, South Africa; ^b^ Africa Centre for Population Health, KwaZulu-Natal, South Africa; ^c^ Institute of Health and Wellbeing, and Royal Hospital for Sick Children, University of Glasgow, Glasgow, UK; ^d^ School of Public Health, Faculty of Health Sciences, University of Witwatersrand, Johannsburg, South Africa

**Keywords:** HIV, adherence, barriers, facilitators, paediatric, anti-retroviral treatment

## Abstract

For children younger than five years, caregivers are responsible for the measurement and administration of antiretroviral medication doses to children. Failure to adhere to the regimen as prescribed may lead to high viral loads (VLs), immune suppression and ultimately drug resistance. In the content of this study, adherence refers to adequate dosing of the medication by a caregiver. Acquired drug resistance to antiretroviral therapy (ART) is prevalent amongst children in South Africa, and poor adherence to the dosing regimen by caregivers may be associated with this problem. In this qualitative study, we purposively recruited 33 caregiver–child dyads from the Hlabisa HIV Treatment and Care Programme database. Children were divided into three groups based on their VL at the time of recruitment. Children with a VL ≥ 400 cps/ml were grouped as unsuppressed (*n* = 11); children with a VL ≤ 400 cps/ml were grouped as suppressed (*n* = 12); and children with no VL data were grouped as newly initiated (*n* = 10). Caregiver–child dyads were visited at their households twice to document, by means of video recording, how treatment was administered to the child. Observational notes and video recordings were entered into ATLAS.ti v 7 and analysed thematically. Results were interpreted through the lens of Ecological Systems Theory and the information–motivation–behavioural skills model was used to understand and reflect on several of the factors influencing adherence within the child’s immediate environment as identified in this study. Thematic video analysis indicated context- and medication-related factors influencing ART adherence. Although the majority of children in this sample took their medicine successfully, caregivers experienced several challenges with the preparation and administration of the medications. In the context of emerging drug resistance, efforts are needed to carefully monitor caregiver knowledge of treatment administration by healthcare workers during monthly clinic visits.

## Introduction

In the context of limited antiretroviral (ARV) drug options available for adults and children in South Africa (Davies et al., 2011), adherence to a first-line regimen is key to ensure optimal and prolonged benefits of treatment (Bangsberg et al., 2000; Bangsberg et al., 2003). According to the latest guidelines on ART initiation among infants, children and adolescents in South Africa, all children younger than five years old should be initiated on ART and are usually initiated on a first-line ART regimen (Department of Health South Africa, 2014). Second-line regimens, although available, remain costly and often difficult to access (Davies et al., 2011). Particular concern has been raised regarding developing resistance to second-line regimens, especially as monitoring of patient viral loads (VLs) is unavailable in many resource-limited settings (Davies et al., 2011; Fox, Ive, Long, Maskew, & Sanne, 2010; Lessells et al., 2014).

For children younger than five years who rely on parental or non-parental caregivers to administer their medication to them daily, adherence becomes an even greater task to manage. In the context of this study, adherence refers to adequate dosing of the medication by a caregiver. In addition to having to provide for children’s most basic needs, caregivers have to integrate and manage complicated regimens into their daily lives (Coetzee, Kagee, & Bland, 2015). For example, in the absence of fixed-dose combinations available to children younger than five years, caregivers of children in this age group are required to carefully and accurately measure and administer volumes of liquid drug formulations to children on ART twice a day (World Health Organisation [WHO], 2013). Moreover, some of the medications have special storage requirements and some are unpalatable and require creative strategies to mask their taste in order to aid administration (Department of Health South Africa, 2014). These factors highlight several characteristics of paediatric ART that complicates adherence.

To our knowledge, no study has observed and documented the barriers and facilitators associated with treatment administration of ART to children younger than five years by their caregivers in their homes during medication times in the morning and evening. The aim of this study was to document, by means of video recording, the barriers to and facilitators of adherence to ART among children younger than five years in rural KwaZulu-Natal.

**Figure 1.**
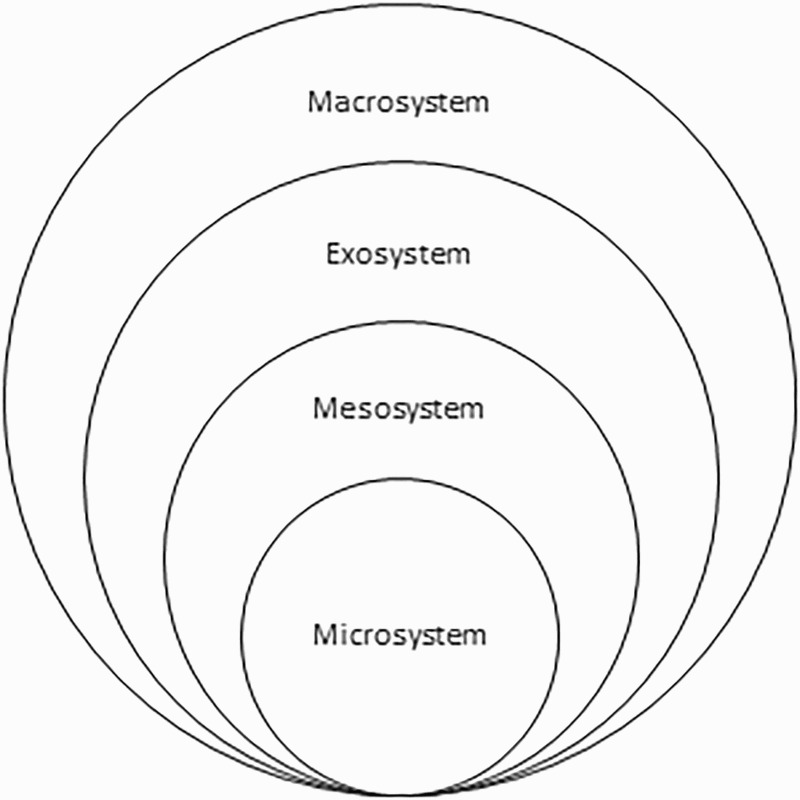
The four systems of Bronfenbrenner’s EST.

This study was conceptualised, interpreted and understood through the lens of Ecological Systems Theory (EST) (Bronfenbrenner, 1979). Ecological theories of health, like EST, consider individual (situated at the micro level) as well as environmental (contextual) factors (meso-, exo- and macro levels) when attempting to understand, or when examining, a target behaviour (see Figure 1). In addition to EST, we used concepts associated with the information–motivation–behavioural skills (IMB) model to discuss and explain specific observed behaviours in this study (Fisher, Fisher, Amico, & Harman, 2006).

## Methods

### Setting

The research was conducted in Northern KwaZulu-Natal (South Africa) under the auspices of the Wellcome Trust-funded Africa Centre for Population Health (www.africacentre.ac.za).

### Participants and procedure

Purposive sampling was used to select and recruit participants from two primary healthcare clinics in the study area. VL status at the time of recruitment was used to differentiate three groups of children invited to take part in the study. Children on ART for more than a year and with two consecutive VLs ≥ 400 copies per millilitre (cps/ml) were grouped as unsuppressed (*n* = 11); children on ART for more than a year with a VL ≤ 400 cps/ml were grouped as suppressed (*n* = 12); and children with no VL data were grouped as newly initiated (*n* = 10).

Adherence counsellors at each of the clinics invited caregivers to meet with the primary investigator (PI) (BC) and research assistant (RA) in a private room after their clinic visit to inform them of the study and to obtain written informed consent for participation. Caregivers were informed that the PI and an isiZulu-speaking RA (to act as an interpreter) were going to visit each of the recruited caregiver–child dyads at their homes twice (one morning visit and one evening visit) to document, by means of video recording, how children received their ARVs. Participants were not excluded if they did not consent to video recording. All participants gave written informed consent, and none objected to video recording. Furthermore, all participants had indicated that household members were aware that the child was taking medications. Following each home visit, participants were handed a food parcel to the value of R150.00 (£6.26) as a token of our appreciation for their willingness to participate and their time in this research.

### Ethics

The study received ethical approval from Stellenbosch University (S12/05/135), permission from the local community advisory board and the hospital management before entering into the field. Multiple visits to participants’ households were used to minimise reactivity bias during observations. Further, both the PI and the RA documented the visits by means of field notes and semi-structured observation schedules, separately. A debriefing session was held after each visit to compare and discuss observations.

### Data analysis

The sources of data were the video recordings, field notes and data from the semi-structured observation schedules used during home visits. Demographic data of the children (such as age, gender, etc.) as well as their caregivers (such as age, marital status, level of education, etc.) were entered and analysed using SPSS v 22. Field notes and video data were analysed thematically using ATLAS.ti v 7 (www.atlasti.com).

## Results

### Child characteristics

The median age of the children at recruitment was 3.5 years (interquartile range (IQR), 2–4.75) for those with suppressed VLs, 4 years (IQR: 3–5) for those with unsuppressed VLs and 2 years (IQR: 2–5) for those newly initiated on ART. Twelve children were between 0 and 2 years old, 5 children were 3 years old, 4 were 4 years old, and 12 were aged 5 years. The majority of children were female (19 out of 33) and most of them (18 out of 33) had their biological mother as primary caregiver. Other primary caregivers were grandmothers (9 out of 33), fathers (1 out of 33) and aunts (5 out of 33). The average time on ART was 2.3 years, 2.8 years and 0.4 years amongst children with suppressed VLs, unsuppressed VLs and those newly initiated, respectively.

### Caregiver characteristics

The median age of the caregivers was 35 years (IQR: 27.5–48), 29 years (IQR: 24–40) and 28.5 (IQR: 25.5–51) for caregivers of children with suppressed VLs, unsuppressed VLs and newly initiated on ART, respectively. A greater proportion of caregivers to children with suppressed VLs (7 out of 12) were married and/or living with a significant other in a permanent union compared to the other two groups (2 out of 11 – unsuppressed, and 2 out of 10 – new enrollers). Furthermore, a greater proportion of caregivers to children with suppressed VLs had completed high school (5 out of 12), compared to the other two groups (2 out of 11 – unsuppressed, and 1 out of 10 – new enrollers). Most of the caregivers (27 out of 33) were unemployed and were receiving either a child support grant or disability grant from the South African government. More than half of the caregivers (19 out of 33) were also ART users. Only 24 (73%) had received the pre-ART HIV education sessions before the child was enrolled on treatment. Most caregivers (25 out of 33) indicated that they were not solely responsible for treatment administration or for attending clinic visits (11 out of 33), but shared this responsibility with another member of the household, usually an older sibling. Caregivers reported that all members of the household were aware that the child was on treatment. However, it was not fully ascertained whether the household members knew this treatment was for HIV, especially in the case of other siblings in the household.

### Context-related observations

#### Medication workspace

The median number of adults and children per household was three. Households had up to six occupants in a dwelling consisting of one bedroom and one living area (approximately 30 m^2^ in total (Hunter & Posel, 2012)). Thus, overcrowded households were common amongst the participants in this study and inhibited the ease with which medication was administered to the child.

#### Medication storage

Nearly all of the caregivers stored the medications in the bedroom. Abacavir (ABC) and Lamivudine (3TC) (both liquid ARV formulations) and tablets (such as Efavirenz (EFV), Zidovudine and Stavudine (d4 T)) do not require refrigeration and it was therefore appropriate to store these medications at room temperature. The directions for one of the medications, Lopinavir/ritonavir (LPV/r), state that it should be kept refrigerated (Department of Health South Africa, 2013). Although most caregivers (30 out of 33) had access to a refrigerator, not all caregivers stored LPV/r in the refrigerator. Compared to children with suppressed VLs, fewer caregivers of children newly initiated and with unsuppressed VLs kept LPV/r refrigerated (Table 1).

**Table 1.** Context-related observations.

	Newly initiated		Suppressed VL		Unsuppressed VL	
	Morning visit (*n* = 10)	Evening visit (*n* = 6)	Morning visit (*n* = 12)	Evening visit (*n* = 8)	Morning visit (*n* = 11)	Evening visit (*n* = 9)
*Medication workspace*						
Lounge	5	4	9	6	2	4
Bedroom	3	2	2	1	7	3
Kitchen	2	0	1	1	2	2
*Medication storage*						
In bedroom (hidden)	5	3	5	3	3	5
In bedroom (not hidden)	2	1	6	5	7	4
In lounge (hidden)	3	2	0	0	1	0
In lounge (not hidden)	0	0	1		0	0
*LPV/r refrigerated*^a^	4 out of 8	2 out of 8	9 out of 10	7 out of 10	5 out of 9	4 out of 9
*Food given*^b^	3	2	1	0	0	0

^a^27 (10 out of 12 suppressed; 9 out of 11 unsuppressed; 8 out of 10 newly initiated) children were receiving LPV/r as part of their regimen.

^b^Food given before treatment administration.

Medications stored in the bedroom were either hidden (when kept in plastic bags and stored in a cupboard in the bedroom), or not hidden (when kept visible on a table in the bedroom). Compared to children with supressed and unsuppressed VLs, more of the caregivers of children who were newly initiated (see Table 1) on ART did not keep LPV/r in the fridge and preferred to store medications out of sight. Based on this observation, it is possible that they were hiding the medications from younger children, other household members or visitors.

#### Food given before treatment administration

During the first visit to the homes, all of the caregivers reported that they usually gave food to the child before treatment administration in the mornings and evenings. However, there were only four morning (three newly initiated, and one suppressed VL) and two evening visits (both newly initiated) in which we had seen a child eating before treatment was administered or had seen visible signs that food had been prepared and given. We did not observe any food given during our visits to the households of children with unsuppressed VLs. There was therefore a mismatch between what caregivers had stated during the initial home visit and what we observed during the morning and evening home visits.

### Medication-related observations

#### Liquid formulations

Apart from one child with a suppressed VL, all of the children received liquid ARV formulations as part of their regimen. For children under three years (<10 kgs), a first-line regimen is a combination of ABC, Lamivudine (3TC) and LPV/r. All three of these medications are available as liquid formulations, and dose changes vary depending on the weight of the child. For children between three and 10 years old (>10 kg), a combination of ABC, 3TC and EFV is recommended (Department of Health South Africa, 2014). Caregivers are required to measure the millilitres prescribed for each medication (usually ranging between 1 and 5 ml) and administer it to the child. Of children newly initiated on ART, 9 received ABC, 10 received 3TC and 8 received LPV/r as part of their regimen. Of children with suppressed VLs, 10 received ABC, 9 received 3TC and 10 received LPV/r as part of their regimen. Of children with unsuppressed VLs, 7 received ABC, 7 received 3TC and 9 received LPV/r as part of their regimen.

The majority of caregivers (observed 20 times during morning administration, and 13 times during evening administration) used a separate syringe to measure and administer each dose (Table 2). One way to aid accurate measurements of liquid formulations using syringes is through the use of a syringe nozzle. Syringe nozzles are small plastic attachments that fit into the opening of medication bottles and are made especially for syringes. These devices are typically packaged with the medications ABC and 3TC. The medication LPV/r does not come with such a device. Syringe nozzles were underutilised amongst the caregivers (only used 15 times across 56 observations) (Table 2). Observation revealed that syringe nozzles were used more frequently by caregivers of children newly initiated (observed six times) and suppressed on ART (observed seven times) compared to unsuppressed children on ART (observed twice). Fewer instances were observed among those with unsuppressed VLs as a large proportion was receiving LPV/r via syringe only.

**Table 2.** Medication-related observations.

	Newly initiated		Suppressed VL		Unsuppressed VL	
	Morning visit (*n* = 10)	Evening visit (*n* = 6)	Morning visit (*n* = 12)	Evening visit (*n* = 8)	Morning visit (*n* = 11)	Evening visit (*n* = 9)
*Medication measurement tools*						
Syringe	6	3	7	5	7	5
Syringe + nozzle	4	2	4	3	1	1
Measuring cup	0	1	1	0	3	3
*Measurement issues*						
Bubble in syringe	1	0	2	1	1	1
*Dose checking*^a^						
ABC	2	3	2	0	4	3
3TC	2	3	1	1	4	3
LPV/r	3	3	8	6	6	7
*Medication administration* (directly or indirectly)^c^						
Syringe (directly)	6	3	9	7	4	4
Measuring cup (indirectly)	4	3	2	1	7	5
*Missed doses*^b^	1	1	0	0	0	0

^a^Dose checking: caregiver checks the measurement dose amount in the syringe for accuracy.

^b^Missed doses: medication is not taken/given on the day.

^c^Medication administration: directly – medication injected into child’s mouth using syringe. Indirectly – medication transferred into a cup.

**Figure 2.**
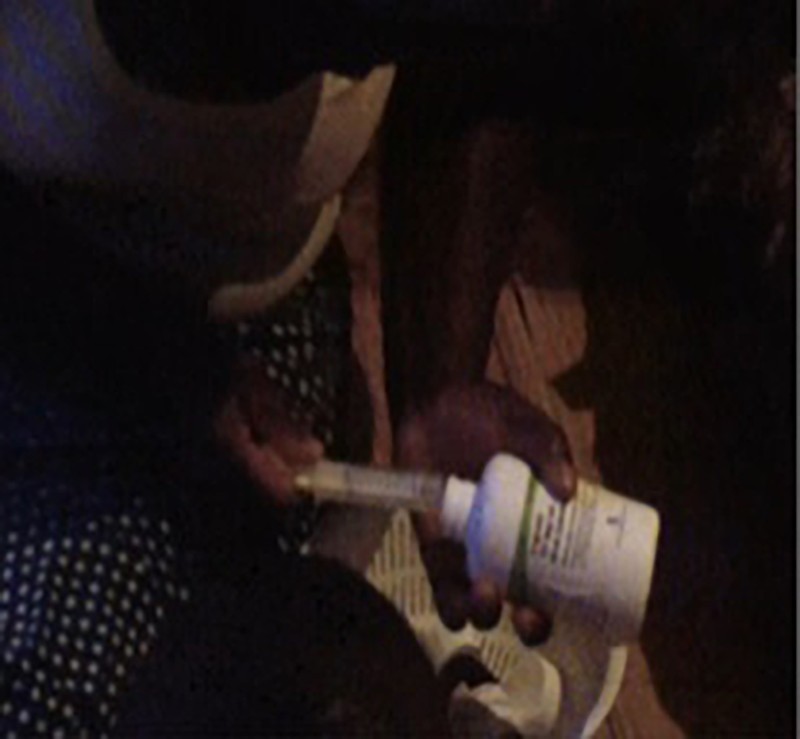
Dose measurement of 3TC taken at an angle. The image illustrates the caregiver of a suppressed child on ART taking a measurement of 3TC at a horizontal angle.

However, even with a syringe nozzle intact, caregivers who chose to use them did so erroneously. In at least two instances where measurements were taken at a horizontal angle, these measurements led to bubble formations in the syringe. Figure 2 shows a caregiver (biological mother) taking a measurement of 3TC with a syringe nozzle intact. The caregiver can be seen holding the bottle horizontally while the measurement is taken. The angle at which the doses were measured resulted in bubbles forming within the syringe, suggesting possible dose inaccuracy. The dose was not checked (for accuracy) by the caregiver after the measurement was made. In four other instances, it was clear from the video data that a large bubble had formed in the syringe (Table 2). In neither of these instances was the bubble removed, indicating possible under-dosing of that medication.

#### Tablet formulations

Tablets and capsules were either swallowed whole or dissolved in water or mixed with a taste distractor (e.g. drinking yoghurt). All the children with suppressed VLs received their tablets/capsules directly and they were subsequently swallowed whole.

The tablets of four out of five children in the unsuppressed group were dissolved in water before being administered. Dissolving tablets into solutions was problematic in cases where the residue of the tablets remained behind in the container (usually a cup) and was discarded by the caregiver when she rinsed out the medication cup. In all four instances where either a tablet or capsule had been dissolved in water, residue of the medications remained behind in glass/cup from which it was administered. Once administered, the caregiver rinsed the administration tool and discarded the residue that remained left over, indicating a possible under-dosing of that medication.

#### Palatability

Most of the children (no problems with palatability in 38 out of 56 observations) ingested their medications without much resistance (Table 3). However, we observed four instances (one newly initiated, one suppressed, two unsuppressed) of children spitting medications out; two instances of vomiting (one newly initiated, one unsuppressed); seven instances of coughing (two newly initiated, four suppressed, one unsuppressed) during medication administration or moaning by the child; five instances of child refusal (one instance in which the child ran away at the time of medication administration (newly initiated); and four instances (two newly initiated and two suppressed) where the child pushed the caregiver’s hand away (Table 3). In all instances of palatability difficulty, the caregiver completed dose administration. In both instances of vomiting in particular, the caregivers only administered a partial dose. As such, children who vomited their medications did not receive their full dosage.

**Table 3.** Child-related observations.

	Newly initiated		Suppressed VL		Unsuppressed VL	
	Morning visit (*n* = 10)	Evening visit (*n* = 6)	Morning visit (*n* = 12)	Evening visit (*n* = 8)	Morning visit (*n* = 11)	Evening visit (*n* = 9)
*Palatability*						
No palatability issues	5	4	10	3	8	8
Spitting	1	0	1	0	2	0
Vomiting	1	0	0	0	0	1
Cough/moans	2	0	0	4	1	0
Refusal	1	2	1	1	0	0
*Taste distracter used (yes)*	0	1	2	2	1	1

## Discussion

To our knowledge, this is the first study to directly observe factors influencing adherence to ART amongst children younger than five years living in a rural area in a resource-constrained environment. Ecological models, like EST, favour behaviour-specific approaches to interventions, such as improving adherence to ART amongst children younger than five years. Thus, by using EST in this study, we were able to identify contextual and medication-related factors influencing adherence to ART among children in a rural area of South Africa.

### Context-related factors

Caregivers in this study showed a preference for keeping medications hidden. Although caregivers were not explicitly asked why they chose to store medications in this way, one might presume that caregivers were hiding the medications from young children or from visitors. Keeping medications hidden adds to the large body of literature on HIV-related stigma (Bogart, Cowgill, & Kennedy, 2008; Brown, Macintyre, & Trujillo, 2003; Katz et al., 2013; Rintamaki, Davis, Skripkauskas, Bennett, & Wolf, 2006; Ware, Wyatt, & Tugenberg, 2006). In this instance, perceived stigma prevented caregivers from accessing cue-based strategies, such as visibility of the medications in their homes, for reminder purposes (Marhefka et al., 2008).

We also observed little to no food administered prior to treatment administration. However, the lack of food and the subsequent administration of medication suggest that caregivers were willing to administer treatment without food. Caregivers’ willingness to adhere to ART in the absence of available resources has often been associated with what Olds, Kiwanuka, Ware, Tsai, and Haberer (2015) have referred to as the “Lazarus effect” (p. 586). In their study, based on the observable improvements in children’s health due to ART use, caregivers of children in rural Uganda were less inclined to miss treatment out of fear that the child may become ill again (Olds et al., 2015). Furthermore, willingness to provide treatment in the absence of food also contradicts evidence from caregivers and healthcare workers. For example, in our previous work, caregivers to children in the same study area as well as doctors, nurses and counsellors reported that lack of food was one of the most important barriers to adherence to ART(Coetzee et al., 2015). While this barrier has been shown to be true in other contexts as well (Haberer & Mellins, 2009; Simoni et al., 2007; Vreeman, Wiehe, Pearce, & Nyandiko, 2008), our data suggest that this does not always mean that doses are missed.

### Medication-related factors

Our results demonstrated that caregivers of children on ART were knowledgeable about some aspects of the medication, but lacked the necessary skills with which to perform accurate measurements of the doses. In several instances, erroneous medication preparation appeared to lead to under-dosing. According to the IMB model (Fisher et al., 2006), information is a necessary condition for behavioural change, but it is not sufficient to bring about that change in itself. Adequate motivation to adhere to the regimen as well as behavioural skills to administer the medication effectively are required for behavioural change to occur. While caregivers used medication tools such as syringes and medication cups to measure doses of the medications, they lacked the necessary skills required for performing accurate measurements, such as dose checking (especially checking that all of the contents of dissolved tablets and capsules were administered) and removing bubbles in syringes. These findings concur with other studies assessing dosing accuracy among caregivers on ART (Howard et al., 2014; Yin et al., 2008).

Caregivers to children living in rural areas have low levels of health literacy (Howard et al., 2014), which may contribute to misunderstandings of how best to administer the medication to children. In other research, we showed that adherence counsellors provided little information to caregivers during monthly clinic sessions, which we attributed to the rushed training that adherence counsellors received and the lack of follow-up and debriefing (Coetzee et al., 2015). However, caregivers to children with suppressed and unsuppressed VLs tended to use devices such as syringe nozzles less frequently than those newly initiated on ART. Thus, carelessness may also be a consequence of treatment fatigue (Bagenda et al., 2011).

While most of the children took their medicines successfully, several still struggled with the palatability of the formulations. In the few instances where vomiting was observed, this may have been attributable to the lack of food in household. Other studies have shown that lack of food before ART is often accompanied by noxious side effects (Biadgilign, Deribew, Amberbir, & Deribe, 2009; Fetzer et al., 2011). Taste-masking and pill-swallowing interventions have contributed suggestions for strategies with which caregivers may deal with these issues (Bain-Brickley, Butler, Kennedy, & Rutherford, 2011). However, these interventions have not been shown to improve adherence to medication in children.

These findings need to be interpreted against several limitations inherent to this research. Although necessary given the methods used, the sample was limited, as only caregivers whose co-household residents knew about the child’s medication taking were recruited. Furthermore, the presence of the PI and RA in participant homes undoubtedly biased some of the observations. Although these findings cannot be generalised, the knowledge is transferable to similar contexts.

### Conclusions

The findings from this research demonstrate the importance of understanding context-specific and age-specific factors that influence adherence to ART among infants and young children. By stratifying these factors according to the micro-, meso-, exo- and macro levels at which they occur, targeted interventions may be developed. To aid accuracy during treatment administration, caregivers’ knowledge of the regimen ought to be monitored regularly by adherence counsellors or relevant healthcare staff. Furthermore, in order to facilitate accurate transfer of knowledge to caregivers, adherence counsellors need to receive regular follow-up training and debriefing sessions. Further research is needed to develop palatable ARV formulations for infants and young children on ART.
